# Elevating the allied health professions workforce: leadership’s role in shaping professional identity

**DOI:** 10.1186/s12913-026-14064-6

**Published:** 2026-02-02

**Authors:** Pippa Hales, Nebil Achour, Olivia King, Hilary Engward

**Affiliations:** 1 Anglia Ruskin University and Royal Papworth Hospital, Cambridge, UK; 2 School of Allied Health, Anglia Ruskin University, East Road, Cambridge, UK; 3 Western Alliance Academic Health Science Centre, Warrnambool, Australia; 4 Veterans and Families Institute for Military Social Research, Anglia Ruskin University, Chelmsford, UK

**Keywords:** Leadership, Allied health personnel, Professional identity, Collective identity, Health workforce, Grounded theory

## Abstract

**Background:**

As global healthcare systems contend with rising pressures, strengthening the Allied Health Professions (AHP) workforce has become a strategic priority. In England, national reforms have promoted AHP leadership to unify a historically fragmented workforce and enhance collective influence. While these reforms have established strategic leadership roles and outlined a national AHP strategy, the impact of AHP leadership on shaping a collective AHP identity remains underexplored. This article addresses this gap by examining how AHP leadership influences the development of a shared identity across the AHP workforce.

**Methods:**

Using a grounded theory methodology, semi-structured interviews were conducted with 22 registered AHPs. Participants represented 11 of the 14 professions recognised as AHPs in the National Health Service (NHS) in England and represented a diversity of experience and health sectors. Constant comparative analysis was used to develop key categories grounded in participants’ perspectives and experiences.

**Results:**

The findings identified four interrelated concepts that explain how AHP leadership enhances or constrains the development of a collective identity: broadening perspective, connecting across AHPs, experiencing inequality within the AHP collective, and experiencing underrepresentation. Findings established that inclusive and visible leadership helped expand awareness beyond individual professions and enabled cross-professional connection. In contrast, inconsistent leadership practices, unequal representation, and the absence of effective, senior AHP leadership reinforce professional silos, undermine belonging, and diminished the perceived value of the AHP collective.

**Conclusions:**

AHP leadership plays a central role in shaping collective professional identity. Where leadership is inclusive, engaged, and prototypical, it promotes cohesion, strategic alignment, and a stronger collective voice. Conversely, limited leadership visibility or inequitable representation exacerbates fragmentation and weakens identity development. To support workforce retention and system impact, policy and practice must prioritise the development of effective, adaptable and inclusive AHP leaders who can create the conditions for meaningful connection, equity, and engagement. These findings offer transferable insights for healthcare systems and a conceptual synthesis of leadership practices that can guide future leadership development and policy.

## Background

Amid growing pressures on global health systems, strengthening the Allied Health Professions (AHP) workforce has become a policy priority [1, 2]. AHPs are a diverse group of healthcare professions who deliver diagnostic, therapeutic, and support services across a wide range of care settings. While definitions vary internationally, they typically include professions such as physiotherapy, occupational therapy (OT), radiography, dietetics, and speech and language therapy, and can also more broadly encompass all registered health professions that are not doctors, nurses, or dentists [3]. In England the AHP workforce includes 14 distinct professions formally recognised by the National Health Service (NHS) [4], making it the third largest clinical workforce in the healthcare system. While AHPs are an established policy and workforce category, it remains unclear how consistently AHP functions as a shared collective identity within an already established workforce grouping.

Despite their size and significance, the AHP workforce has historically been fragmented, lacking shared infrastructure, unified leadership, or a collective voice. In response to this fragmentation, countries such as England, Australia, and New Zealand have sought to unite the AHP workforce through strategic leadership roles, with the appointment of Chief Allied Health Professional Officers (CAHPO) in England and New Zealand, and the establishment of a national confederation of AHPs in Australia. In England, the appointment of the CAHPO and the launch of a national AHP strategy [4] marked a pivotal step toward professional cohesion and clarifying the workforce’s contribution to evolving healthcare demands. These reforms placed AHP leadership at the centre of efforts to drive influence, integration, and system-wide impact, aiming to release the workforce from its historical boundaries.

Policy reforms in England have expanded AHP leadership roles and promoted collective recognition [5]. However, literature on AHP leadership focuses primarily on organisational structures, with limited attention to workforce experience [6, 7]. Studies from Australia, such as Boyce and Jackway [8] and Mickan, Dawber and Hulcombe [9], highlight the under-representation of AHPs in senior decision-making roles and advocate for stronger, more unified leadership to improve service outcomes and patient safety. While these accounts advance the case for strategic leadership by emphasising benefits such as visibility and alignment with organisational goals, they rarely interrogate the cultural or relational mechanisms through which leadership influence’s identity, belonging, or inter-professional cohesion.

To fully interrogate this gap, it is helpful to explore how professional and collective identity are conceptualised in the literature, to provide context for the study setting and to highlight the conceptual complexity of AHP identity, rather than to serve as an a priori analytic framework. The concept of professional identity, how individuals perceive themselves in relation to their work and peers, has been extensively theorised within sociological and organisational literature [10–12]. For AHPs however, the picture is more complex as their identity operates on multiple levels. First, as members of distinct uni-professions, shaped by formal training, regulatory standards, and professional values [13, 14], and second, as part of a broader collective that brings together the diverse professions under a shared identity [7]. Collective identity in this context draws from work in social movement theory [15], referring to a sense of alignment with a broader professional group that transcends individual disciplinary boundaries [16]. This framing acknowledges that identity is shaped not only by formal policy and leadership structures, but also by individuals’ experiences of their place within the system. Leadership’s role in shaping collective identity is therefore critical to strengthening cohesion, engagement, and strategic impact across the AHP workforce. However, there remains limited empirical evidence examining how AHP leadership is experienced across professions and how it shapes collective AHP identity in everyday practice.

Zwolsman’s [17] work begins to bridge the structural and experiential divide by exploring how AHP leaders position themselves during system reform, revealing tensions between subordinate and self-authorised leadership identities. Yet even this more relational analysis remains focused on those already in leadership roles, offering limited insight into how leadership frameworks shape identity and belonging across the wider AHP workforce. Understanding how leadership influences collective identity is therefore critical to strengthening cohesion, engagement, and the strategic potential of AHPs within healthcare systems.

In England, the evidence base on AHP leadership remains limited and largely shaped by policy rather than empirical investigation [4, 5, 18]. Strategic guidance may emphasise the importance of collective AHP leadership for cohesion and influence, but there is a striking absence of studies examining whether such leadership is felt, recognised, or effective in shaping a shared sense of identity among AHPs. A recent study by Mizzi and Marshall [19], exploring the experiences of AHPs in England, provides empirical insight into the persistent inequalities in access to leadership and professional development. While their work begins to illuminate the experience of AHPs in the NHS in England, it stops short of exploring how leadership dynamics shape collective professional identity. The connection between leadership and collective identity formation remains critically underexplored.

This article addresses that gap by examining how AHP leadership intersects with the development of a collective AHP identity in the NHS in England. It offers empirical insight into how leadership is experienced across professions, how it may support or constrain a more connected and strategically influential workforce and identifies leadership practices to guide future practice and policy.

## Methods

### Research design

This paper presents findings from a broader study that explored the development of a collective AHP identity within the NHS in England, drawing on the experiences and perspectives of AHPs. While the broader research addressed various factors shaping identity, this article focuses specifically on the role of AHP leadership, which was identified as a central category, warranting in-depth analysis.

A grounded theory methodology was selected for its suitability in exploring areas where little is known, and for its ability to generate theory grounded in participants’ experiences [20, 21]. This made it particularly appropriate for investigating collective AHP identity, which remains underexplored in existing literature. In the context of limited empirical research on how AHPs themselves experience these issues, a bottom-up approach was essential to ensure that participants’ perspectives shaped the development of theory. A constructivist lens to grounded theory [22], was applied to reflect the co-constructed nature of the research process and to acknowledge the researcher’s positionality. Consistent with a constructivist grounded theory approach, we did not apply an a priori theoretical framework to the data. Categories were constructed through iterative engagement with participants’ accounts and constant comparison, with existing literature used to contextualise and refine interpretation at later analytic stages. The primary researcher, a practicing AHP within the NHS, used reflexive strategies throughout to critically examine their influence [23], ensuring that participants’ voices remained central to the theory-building process.

### Sampling and recruitment

Participants were purposively sampled, using demographic data, to recruit AHPs working clinically in the NHS in England. Those working exclusively in non-clinical leadership roles or outside of the NHS in England were excluded to ensure that the data reflected the direct, on-the-ground experiences of clinical AHPs. Including individuals in strategic leadership positions could have introduced bias, as their advanced knowledge of the AHP collective and existing leadership strategies may have influenced the development of categories. This sampling decision was intended to centre the experiences of clinical AHPs and minimise the risk that strategic or policy-level interpretations of the AHP collective would dominate category construction. Accordingly, our leadership findings reflect how leadership is experienced by clinical staff in relation to collective identity formation. Recruitment was conducted via social media, chosen to reach a national audience efficiently and maximise breadth across the 14 AHP professions. Informed consent was obtained from all participants prior to their interviews. In accordance with grounded theory methods, participant recruitment, and constant comparative data collection and analysis continued until theoretical sufficiency was achieved, defined as the point at which categories were well-developed and additional data no longer contributed new insights relevant to the research aim [24].

### Data collection and analysis

Data were collected through semi-structured, one-to-one interviews, conducted by the primary researcher, via Microsoft Teams™. Semi-structured, individual interviews were selected to allow participants to speak freely about their experiences, while minimising the social desirability bias that can occur in group settings [25]. An interview guide was developed for the study and used to support the semi-structured interviews and was refined iteratively throughout the concurrent processes of data collection and analysis. Interviews were recorded, transcribed verbatim, and adapted iteratively based on early code and category development, in keeping with grounded theory’s dynamic and recursive design. As leadership became increasingly prominent in participants’ accounts, later interviews used open-ended prompts focused on what shaped inclusion within the AHP collective. This enabled further exploration of leadership visibility, inclusive or exclusionary practices, and issues of representation across professions.

Data collection and analysis proceeded concurrently. Initial coding was conducted line-by-line to remain grounded in participants’ words, followed by focused coding to identify and develop conceptual patterns [22]. Analytic memo-writing supported iterative refinement as patterns across professions and contexts became clearer. This process constructed categories capturing key influences on the development of a collective AHP identity, with AHP leadership identified as a prominent and analytically developed category. This article presents findings related to that category, using participants’ first-hand accounts to construct a theoretically grounded explanation of how leadership shapes collective AHP identity formation.

### Ethical considerations

The study received ethical approval from the Anglia Ruskin University School of Allied Health and Medicine Research Ethics Panel, and was ratified by the Faculty of Health, Education, Medicine and Social Care Research Ethics Panel in August 2021 (AH-SREP-20-150).

### Ensuring rigour and quality

Rigour and quality were ensured through methodological practices being aligned with grounded theory, including the systematic application of its core methods [20] and ongoing reflexivity [26]. Analytical memos and diagramming were used to trace the construction of categories and their development into conceptual insights to support dependability and confirmability [27]. Credibility was enhanced through the iterative nature of grounded theory, which ensured that developing categories were grounded in participants’ experiences and reflected conceptual resonance.

## Results

A total of 22 participants were recruited, representing 11 of the 14 AHP professions, formally recognised in the NHS in England. The sample included: occupational therapists [4], physiotherapists [3], speech and language therapists [1], diagnostic radiographers [2], art therapists [2], operating department practitioners [2], dietitians [3], podiatrists [1], paramedics [1], orthotists [1], and music therapists [2]. Participants had a range of clinical experience, with 10 registered for five years or less and 12 registered for over five years, providing a balance of early-career and more experienced practitioners. Interviews ranged between 50 and 75 min each. Through iterative coding and constant comparison, four interrelated concepts were constructed that explain how participants experienced AHP leadership as shaping collective AHP identity. These were: broadening perspective, connecting across AHPs, experiencing inequality within the AHP collective and experiencing underrepresentation.

### Broadening perspective

This concept captures how leadership influences AHPs’ ability to expand their perspective beyond their registered professional role. Participants described how early career AHPs typically concentrate on mastering the clinical and technical aspects of their own profession.

I think you get very tunnel visioned as a fresh graduate. In the first job you just think ‘right, OK, I need to know who to go to and I’m just treating this part of the body. (Participant 10)

While such a focus is understandable at the outset, leadership was identified as a key factor in purposefully broadening this view, encouraging awareness of other AHP roles, the interdependence of care, and a sense of belonging to a wider professional community.

One participant in a senior role described a staged, uni-profession-centred model of early development, aligned with NHS bands, a pay and seniority grading system used across the NHS AHP workforce:

For a band five, I want them to worry about their bit of work. For a six, I want them to worry about their work and a bit of somebody else and supervising somebody else and maybe what the physio is doing. (Participant 3)

While participants recognised the value of a staged approach for building uni-profession skills and confidence, they also suggested that it could reinforce early profession-specific boundaries, limit opportunities for interprofessional working, delay wider awareness of other AHP roles, and weaken connection to the AHP collective. Participants emphasised that leadership behaviours shape how and when practitioners move from a primarily uni-profession focus to a broader AHP perspective. However, the opportunity to broaden one’s view was often absent or inconsistent:

We don’t have those [meetings with other AHPs] at Band 5 and Band 6. It’s purely at management level, there’s no interlinking at a lower level. (Participant 7)

This AHP illustrates how early career AHPs experience exclusion from cross-professional engagement, reinforcing siloed ways of working. While perspective may broaden with experience, the findings suggest it does not happen organically for all.

Without deliberate efforts from leaders to facilitate cross-professional interaction and reinforce a shared AHP identity, many participants reported remaining focused solely on their own profession. Broadening perspective, then, is not simply a matter of time or exposure, it is a developmental process that depends on intentional, inclusive leadership from the outset.

### Connecting across AHPs

This concept captures how leadership creates or fails to create opportunities for AHPs to engage meaningfully across uni-professional boundaries. While participants valued the idea of being part of a wider AHP community, they described a lack of organic connection between professions. Most did not view it as their responsibility to initiate collaboration, instead looking to leadership to coordinate collective spaces and relationships.

We’re very much focused on promoting our own role, than the AHP. (Participant 8)

This comment reflects a dominant theme of AHPs primarily working within their own professions, with little impetus to step outside that frame unless prompted by external structures. Participants pointed to leadership at both local and national levels as responsible for facilitating these broader professional connections, yet described such leadership as inconsistent and, at times, absent.

We’ve got maybe eight or nine of the AHP professions, but actually we don’t have any official person that pulls us together. (Participant 3)

Without someone actively bringing professions together, the AHP community was described as fragmented. The presence or absence of a lead AHP shaped whether participants felt part of a wider AHP collective. Of the 22 participants, 10 confirmed their organisation had an AHP lead, 3 said there was none, and 7 were unsure. Notably, 6 of the 7 who were unsure were early career AHPs who did not express having an AHP identity, suggesting a link between limited cross-professional exposure and limited collective identity.

One early career AHP, without an AHP identity, was asked if there was an AHP lead in their organisation. They responded:

I don’t. I’ve not heard of one. I think I would have probably noticed. (Participant 15)

Later checks confirmed that the organisation did, in fact, have a lead AHP. This highlights how disconnection from leadership can mirror a lack of identification with the collective. Conversely, participants who had experienced engaged AHP leadership described its unifying and empowering effects:

She [the Chief AHP] came and found us and gave us all of these [AHP] badges, so that was part of it, was kind of being drawn in by the lead AHP in the organisation and kind of making friends. (Participant 1)

For others, simply knowing that an AHP leader existed offered reassurance:

It feels good to have that Chief AHP hidden in our corner for that fight. He’s making waves and it feels really nice to have that voice for us. (Participant 19)

Together, these accounts suggest that leadership plays a pivotal role in helping AHPs connect beyond their individual professions. Without proactive, visible leadership to create these connections, the collective identity struggles to form, and the benefits of belonging to a unified AHP workforce remain out of reach.

### Experiencing inequality within the AHP collective

This concept captures how internal inequalities within the AHP group can undermine collective identity and belonging and the importance of AHP leadership in mitigating this. Participants described unequal representation across professions as a persistent issue, often reinforced by leadership practices, either through oversight or a narrow focus on certain dominant professions.

While AHP leadership was broadly viewed as beneficial, several participants reflected on how it can unintentionally marginalise certain professions:

We often get a bit missed out of the AHP work. Our lead AHP, she’s lovely, but in her head it’s the occupational therapists and the physios and then she kind of goes ‘Oh yeah, there’s you there. (Participant 1)

These comments reflect how exclusion from AHP initiatives and discourse was experienced by some participants as causing individuals and their uni-professions to feel disconnected from the collective. The experience of being overlooked was described as reinforcing a sense of isolation and was associated with a weaker connection to a shared AHP identity.

Participants from smaller professions particularly highlighted this imbalance. They described how leadership roles were held by members of larger or more established professions. This lack of representation in leadership was perceived to reinforce internal inequalities within the AHP collective:

If you look at the higher bands and the leaders in AHP, there’s not going to be a podiatrist there. There’s a lot of physios, a lot of you know, there’s OTs [occupational therapists], but podiatry, because we’re such a small group we’re not represented. (Participant 17)

Without inclusive leadership that understands and actively advocates for the full breadth of AHP roles, participants suggested these inequalities were perpetuated rather than addressed.

This perception of unequal visibility was compounded by inconsistent efforts from leadership to understand the breadth of roles within the AHP collective. Participants stressed the importance of leaders engaging meaningfully with all professions:

It’s important they [the Chief AHP] make every effort to understand the individual issues of the individual disciplines and to not make assumptions that everyone’s the same. (Participant 9)

When leaders did make the effort, it was noticed and valued. One participant described a positive example of an AHP lead addressing their own knowledge gap:

The other week I had the Trust AHP lead, who’s a physio, come to do a day with me because she’s got no idea what an ODP [operating department practitioner] does. (Participant 20)

Such actions signalled genuine interest and were seen as steps toward building a more inclusive and cohesive collective identity.

Participants emphasised that leadership in this context involved inclusion and equitable representation as well as coordination. Where inclusion across professions was inconsistent, participants described the AHP identity as fragmented. These experiences indicate that addressing internal inequality is important for sustaining a collective identity where every profession feels seen, valued, and part of the whole.

### Experiencing underrepresentation

This concept captures how the absence of visible and influential AHP leadership, particularly at senior levels, reinforces a sense of the uni-professions within the AHP collective being overlooked and undervalued. Participants described how AHPs often lacked representation in key organisational forums, with other professional groups positioned to speak on their behalf.

While participants were often grateful for support from colleagues in other professions, they questioned whether their interests could be effectively championed without direct AHP representation:

We’re [AHPs] still outnumbered by senior leaders from other professional groups and that resonates on the ground. (Participant 5)

This concern was reinforced by perceptions that even when AHP leaders were present, they occupied less influential positions than their counterparts in other professions:

We have an AHP lead here within the Trust and she’s tremendously supportive. But she doesn’t sit on the board the same way that the nurse lead does. (Participant 20)

This absence from senior forums contributed to a perception that AHPs and their contributions were not fully recognised or prioritised within wider organisational agendas. The consequence of this, as one participant explained, was a sense that their patients and professions were left without a voice:

It [no AHP lead] means that we’re not always at the table and I guess at the end of the day I kind of feel that my patients aren’t represented, that part of their world isn’t represented at the table. (Participant 6)

There was also frustration at the default assumption that nurses could adequately represent the diverse range of AHP roles. While participants acknowledged nurses’ efforts, they highlighted the limitations of such cross-professional representation:

The nursing director definitely tries, but they’re so swamped with other stuff that knowing the intricate workings of 14 other professional groups I wouldn’t say was very high on their agenda at all. (Participant 5)

These experiences reveal a deeper tension between advocacy and understanding. Without direct AHP representation in decision-making spaces, participants felt their professions were underrepresented. They expressed a strong desire for leadership that could understand, articulate, and advocate for their professions from a position of lived experience:

Why can there be a medical lead on the board and a nurse lead on the board, but there’s not an AHP lead on the board, when we have an AHP lead? (Participant 6)

Across the data, participants identified AHP leadership as important in raising the profile of professions within the AHP group, supporting inclusion in organisational decision-making, and advocating for the value they bring to patient care. The absence of this leadership, or the substitution of it, was experienced not only as a lack of professional recognition but as a structural gap that undermined the integrity of the AHP collective identity.

## Discussion

The findings demonstrate that AHP leadership plays a central role in shaping collective AHP identity through four interrelated concepts: Broadening Perspective, Connecting Across AHPs, Experiencing Inequality Within the AHP Collective, and Experiencing Underrepresentation. Leadership was seen to both enable and constrain identity development. Effective, inclusive, visible leaders supported connection and belonging, while absent or ineffective leadership reinforced fragmentation and marginalisation, particularly for smaller professions. These findings suggest that the development of a collective AHP identity is not automatic but shaped by the conditions leaders create for connection, recognition, and participation. Consistent with grounded theory, existing identity and leadership frameworks are used here to place these data-driven categories in dialogue with broader scholarship. We first interpret these findings in relation to identity and belonging, then consider the conditions that shape identity clarity and inclusion across professions, before addressing structural constraints and implications for leadership development.

### Role and collective identity within the AHP category

Our findings highlight an interplay between strong profession-specific role identities and a broader, more variably salient AHP collective identity. Although the AHP category is well-established administratively, participants’ accounts suggest that its significance for self-definition remains inconsistent. This pattern highlights that collective AHP identity is not simply conferred by policy designation but is constructed through local and system-level leadership, narratives of shared contribution, and experiences of inclusion and recognition. This also helps explain how AHP leadership may support identity motives such as distinctiveness and continuity while enabling clinicians to maintain strong profession-specific identities within a broader collective.

This pattern can be placed in dialogue with Identity Theory, which distinguishes personal, role, group and social category identities and emphasises that identities vary in salience, centrality and commitment across contexts [28]. In our data, profession-specific role identities appear highly stable and central to day-to-day practice, while the AHP collective identity is more variable and shaped by leadership conditions that enable recognition, inclusion and cross-professional connection.

### Leadership, belonging and cross-professional connection

The findings reinforce the importance of leadership in building and sustaining the collective AHP identity. While national strategies emphasise the importance of collaborative and transformational leadership in addressing NHS workforce challenges [1, 29, 30], this study reveals how, in practice, the presence or absence of effective AHP leadership directly influences whether a shared identity is meaningful, inclusive, or empowering. Participants described leadership as both an enabler and a barrier: skilled, visible leaders created connection and belonging across professions, while ineffective or absent leadership reinforced fragmentation, particularly for smaller or marginalised AHP groups. These findings highlight the need for intentional leadership that not only represents the collective but actively nurtures its development and sustainability.

To interpret the identity-related conditions described by participants, we place our four concepts in dialogue with Allen et al.’s [30] framework on belonging. The concepts of Connecting across AHPs and Broadening Perspective, point to a critical leadership responsibility: enabling meaningful interprofessional engagement that supports identity formation beyond one’s own uni-profession. This aligns with Allen et al.’s [31] framework on belonging, which identifies four key components; competence, opportunity, motivation, and perception, as necessary conditions for cultivating a sense of group belonging. While these dimensions were not explicitly referenced by participants they were reflected in their experiences. Participants generally demonstrated the competence and motivation to form interprofessional connections, yet early career structures were often narrowly focused, limiting these opportunities unless leaders intervened. Opportunities to connect were seen as dependant on leaders establishing inclusive, cross-professional forums, efforts that participants described as inconsistent or absent. Motivation to identify with the AHP collective fluctuated in response to leadership behaviours: when leadership marginalised certain professions or failed to promote equity, participants reported disengagement and a weakened sense of collective identity. Finally, perceptions of belonging were strongly linked to leadership visibility and influence; where AHP leaders were absent or lacked authority, the AHP identity was experienced as superficial or imposed rather than empowering and meaningful.

We recognise that belonging is one important dimension of identity construction and that clinicians also draw strongly on profession-specific role identities in everyday practice. Participants in this study frequently described deep attachment to their uni-professions, which often provided a stable basis for self-definition and professional meaning. However, their accounts suggest that a collective AHP identity became more salient in contexts where cross-professional inclusion, visibility and influence were at stake, particularly for smaller professions. This helps explain why leadership practices that create consistent opportunities for connection and equitable representation remain important even in the presence of strong profession-specific identities.

Taking the study findings together with Allen et al.’s framework, it highlights that cultivating a sense of belonging within the AHP collective depends not only on structural initiatives but also on relational and representational leadership practices. Without consistent, inclusive leadership practices, the conditions for belonging to the AHP collective risk being unmet, particularly for smaller or marginalised professions. This points to a leadership development gap: it is not enough to train leaders in policy implementation or strategic oversight; they must also be equipped to understand and act on the social dynamics of identity formation. Embedding this understanding into leadership frameworks and training could help ensure that the collective AHP identity is not only promoted at a national level but also experienced meaningfully across clinical settings.

### AHP identity clarity and leadership visibility

In this study, participants described a lack of clarity around the collective AHP identity and its relevance to their professional experience. This was particularly evident when participants were unsure whether an AHP leader existed in their organisation or described minimal engagement with AHP-focused initiatives. Such disconnection weakened their sense of belonging and limited their perceived value of being part of a collective. Addressing this requires clearer, more consistent communication from leadership about the purpose of the AHP identity and the role it plays across all professions. This supports Haslam, Reicher and Platow’s [32] view that leadership is not shaped by group identity, but rather leadership must actively shape group identity. While the National AHP Strategy [1] sets out a national definition of the AHP collective, these findings reveal a gap between strategic messaging and how AHPs experience leadership and identity formation in practice.

### Inclusion, negotiated meaning and identity dissonance

Participants’ accounts suggest that the meaning of AHP remains uneven and locally negotiated across professions and organisations. In this context, leadership that is trust-building and integrative becomes particularly important, not to override uni-professional identities, but to enable a workable shared narrative, equitable representation, and cross-professional inclusion. This interpretation resonates with Rhee et al.’s [33] category scholarship, suggesting that when agreement about meaning is limited and authority to stabilise meaning is decentralised, the durability of a category may depend on negotiation and compromise between the professions it brings together. This helps explain why inclusive, trust-building leadership may be especially important at this stage of collective AHP identity construction.

The findings further suggest that where leadership fails to explicitly value and include smaller or less prominent professions, identity dissonance may develop. Drawing on Hogg and Rast’s [34] Intergroup Relational Theory, this dissonance can be seen as a failure to build a shared identity that respects and incorporates the distinctiveness of each uni-profession. Rather than erasing difference, which can lead to threat and conflict, effective AHP leadership must acknowledge and elevate the uni-professions, reframing the collective as a space of inclusion and mutual reinforcement. The AHPs’ experience in this study reveals the need for targeted leadership development and clear guidance to equip AHP leaders with the skills and strategies necessary to actively and equitably represent their workforce. By effectively advocating for the uni-professions and promoting the mutually beneficial relationship between uni-profession and collective AHP identities, leaders can then create a unified and effective workforce, address identity dissonance and mitigate the risk of sustained fragmentation.

### Structural constraints on AHP leadership and representation

While the findings emphasise the influence of AHP leadership roles, participants’ reflections on the limited visibility, influence, and inclusivity of senior AHP leadership closely mirror patterns identified in existing literature. The small but growing body of empirical work on AHP leadership highlights that these roles are themselves affected by structural inequality [17, 19, 35, 36]. These studies report inconsistencies in the presence of strategic AHP positions and status, both in England and internationally. They also underline the benefits of profession diversity at senior levels while recognising that historical and institutional healthcare structures continue to act as barriers to achieving this. Mizzi and Marshall’s [19] study on barriers and opportunities for AHP leadership in the NHS in England reinforces this view, identifying a lack of equitable leadership development opportunities as a key challenge. Such barriers not only constrain individual career progression but also perpetuate the same inequalities and undervaluation that AHP leadership is expected to redress, ultimately limiting its ability to exert meaningful influence across healthcare systems.

### Implications for leadership development and policy

Addressing these issues requires recognising the importance of AHP leadership positions being held by AHPs themselves. Social Identity Theory [37] stresses that effective leaders must be prototypical, exemplifying the group’s values and shared purpose to influence its members into social action [32, 38]. This principle aligns with the findings of this study, which show that AHPs view the presence of AHP leaders, those who embody the group values as an AHP, as a positive force. Conversely, the absence of such leaders is experienced negatively, reinforcing the importance of visible, prototypical leadership by AHPs for AHPs. These insights indicate the need for leadership development opportunities and systemic changes to ensure that AHPs are represented by prototypical leaders who can unify the workforce and inspire collective action to enhance patient outcomes.

In addition to social identity leadership, it may also be valuable to consider the role of leader identity in AHP leadership development. Haslam et al. [39] highlight that leaders’ self-understanding of who they are as a leader interacts with their capacity to enact identity-relevant leadership for others. In the present study, participants’ accounts emphasised the importance of visible, inclusive and representative AHP leadership; strengthening leader identity as an AHP leader may therefore support leaders to more confidently articulate a shared narrative, advocate equitably across professions and create the conditions for cross-professional inclusion. Future research could usefully explore how AHP leaders’ self-concepts align with, shape or sometimes diverge from clinical AHP experiences of collective identity formation.

Recognising the complexity of healthcare systems, it is important to acknowledge that AHPs exist within a shifting and multifaceted healthcare structure. As such, leadership must be agile and responsive to these ongoing shifts [19, 29]. AHP leaders must therefore stay attuned to the ongoing changes in healthcare, recognising that the collective identity must evolve alongside these shifts as part of the professions’ historical continuum, while also understanding the crucial role they play in shaping and influencing these changes. Effective AHP leadership should embrace adaptability and encourage inter-professional collaboration, ensuring that the collective identity strengthens integration rather than isolation. Future policies and initiatives should therefore support the development of these adaptive leadership skills, emphasising the importance of collaboration and inclusivity to prevent the formation of AHP as a professional silo and ensure alignment with the evolving demands of healthcare.

**Fig. 1:**
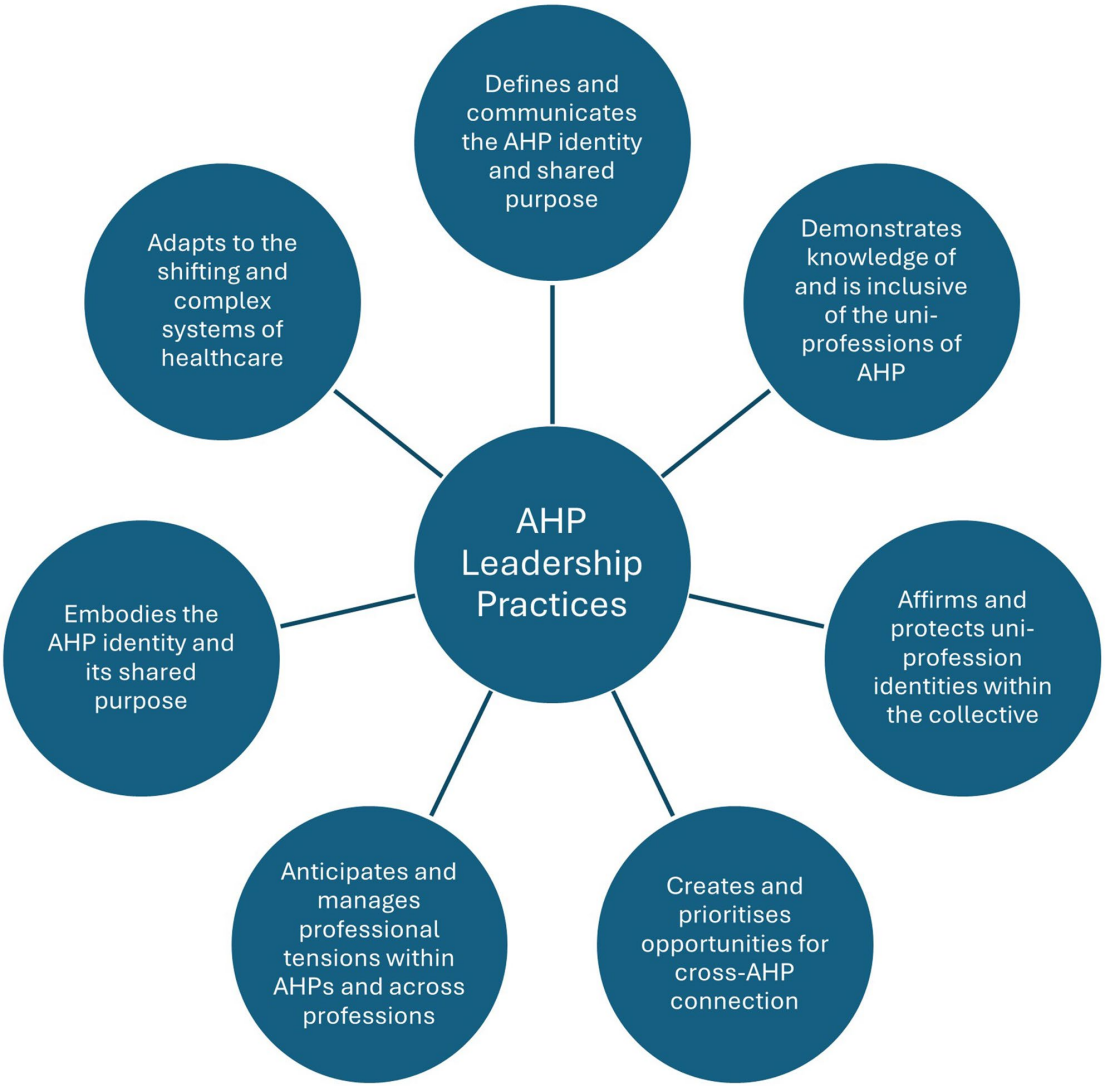
Conceptual synthesis of leadership practices required to support collective AHP identity in the NHS. Source: developed from participant insights and existing leadership literature

Drawing on the study findings and existing leadership literature, this study presents the leadership practices essential to support the development of a collective AHP identity in the NHS (Fig. 1). These practices are not presented as new empirical data, but as a conceptual synthesis informed by participants’ perspectives and aligned with established literature. We recognise that these practices do not place equal expectations on all leaders. System-level and organisational AHP leaders are more likely to shape the collective narrative, visibility and cross-professional infrastructure that enable identification at scale, whereas local leaders and direct supervisors can most realistically influence the day-to-day conditions of inclusion, recognition and cross-professional respect. This figure offers a practical lens for future leadership development and policy.

### Strengths, limitations and future research

A key strength of this study is its inclusion of participants from a wide range of AHP professions and levels of experience. The sample included early-career and senior clinicians, offering insight into how leadership is experienced across different career stages and professional contexts. This diversity enhances the relevance and resonance of the findings across the AHP workforce.

However, with 11 of the 14 professions represented, it does not include orthoptists, osteopaths, or dramatherapists, professions that may face distinct challenges not fully captured here. While the findings offer a strong representation of the AHP collective, further research is needed to ensure all professions are equitably understood and represented.

Future research should explore how collective AHP identity influences workforce outcomes, particularly retention and attrition. Understanding the relationship between leadership, identity, and workforce sustainability will be key to shaping effective strategies that support a connected, engaged, and resilient AHP workforce. Future research could also explore how AHP leaders’ self-concepts align with, shape or sometimes diverge from clinical AHP experiences of collective identity formation, and what this means for leadership development.

## Conclusions

This study highlights the pivotal role of leadership in developing and sustaining a strong collective AHP identity. Effective AHP leaders facilitate professional connection, promote inclusion, and support the elevation of uni-profession identities within a shared collective purpose. In contrast, leadership that lacks visibility, equity, or clarity can exacerbate fragmentation, undermine belonging, and limit the influence of AHPs across the healthcare system.

To address ongoing workforce challenges, policy and practice must prioritise the development of skilled, adaptable AHP leaders who can nurture cohesion, advocate for all professions within the collective, and navigate the complexities of a changing healthcare system. Clearer communication of the collective AHP purpose, equitable leadership development opportunities, and inclusive representation across all professions are essential next steps. Strengthening AHP leadership in this way offers a powerful route to enhancing the overall impact of the AHP workforce on patient outcomes and system-wide transformation.

## Data Availability

The datasets are not publicly available due to the nature of qualitative data and participant confidentiality. An initial version of the semi-structured interview guide is available from the corresponding author upon reasonable request.

## Abbreviations

AHP: Allied Health Profession/al
AHPs: Allied Health Professions/als
NHS: National Health Service
OT: Occupational Therapist
ODP: Operating Department Practitioner
